# Identification of a novel glycolysis-related gene signature for predicting the prognosis of osteosarcoma patients

**DOI:** 10.18632/aging.202958

**Published:** 2021-05-05

**Authors:** Mengkai Yang, Xiaojun Ma, Zhuoying Wang, Tao Zhang, Yingqi Hua, Zhengdong Cai

**Affiliations:** 1 Department of Orthopedics, Shanghai Bone Tumor Institution, Shanghai General Hospital, Shanghai Jiao Tong University School of Medicine, Shanghai 200080, P.R. China

**Keywords:** osteosarcoma, glycolysis, metastasis, prognosis, P4HA1

## Abstract

Glycolysis ensures energy supply to cancer cells, thereby facilitating tumor progression. Here, we identified glycolysis-related genes that could predict the prognosis of patients with osteosarcoma. We examined 198 glycolysis-related genes that showed differential expression in metastatic and non-metastatic osteosarcoma samples in the TARGET database, and identified three genes (P4HA1, ABCB6, and STC2) for the establishment of a risk signature. Based on the signature, patients in the high-risk group had poor outcomes. An independent Gene Expression Omnibus database GSE21257 was selected as the validation cohort. Receiver operating characteristic curve analysis was performed and the accuracy of predicting the 1- and 3-year survival rates was shown by the areas under the curve. The results were 0.884 and 0.790 in the TARGET database, and 0.740 and 0.759 in the GSE21257, respectively. Furthermore, we applied ESTIMATE algorithm and performed single sample gene set enrichment analysis to compare tumor immunity between high- and low-risk groups. We found that the low-risk group had higher immune scores and immune infiltration levels than the high-risk group. Finally, we chose P4HA1 as a representative gene to verify the function of risk genes *in vitro* and *in vivo* and found that P4HA1 could promote the metastasis of osteosarcoma cells. Our study established a novel glycolysis-related risk signature that could predict the prognosis of patients with osteosarcoma.

## INTRODUCTION

Osteosarcoma (OS) is a primary malignant tumor that mainly occurs in adolescents and young adults [1]. When lung metastasis occurs, the 5-year survival rate of these patients is less than 30 % [2]. Although the treatment of OS, which includes a combination of surgical excision and systemic chemotherapy or radiotherapy, has greatly improved, only 11–30 % of the patients presenting with metastatic OS survive after the combination therapy [3, 4]. Therefore, there is a need to explore and discover potential new therapeutic targets and biomarkers for OS patients with metastasis.

Metabolic reprogramming is an important hallmark of cancer [5]. Aerobic glycolysis, also called the “Warburg effect,” is best known as a form of metabolic reprogramming that occurs in virtually all cancer cells. Tumor aerobic glycolysis can be a good indicator in the prognosis of cancer [6] and is a promising therapeutic target for tumor therapy based on the clinical observation of a significant increase in glucose uptake in tumor tissue relative to that in adjacent normal tissue [7]. Glycolysis also regulates the progression of metastasis in many cancer cells; the metastasizing cells become dependent on glycolysis for adenosine triphosphate (ATP) production. For example, Yang et al. showed that enhancement of glycolysis promotes the metastasis of pancreatic cancer cells [8], whereas in oral squamous cell carcinoma, the novel lncRNA lnc-p23154 accelerates the metastatic potential of cancer cells through GLUT1-mediated glycolysis [9]. Recently, many studies have demonstrated that glycolysis also promotes the progression of OS. Glycolysis is reportedly involved in the process of taurine-upregulated gene 1 (TUG1)-induced growth in OS [10], and restriction of glycolysis inhibits the metastasis of OS, both *in vivo* and *in vitro* [11]. Consequently, elucidation of the relationship between glycolysis and metastasis in OS is required, since better understanding of the glycolysis-mediated alterations in OS could help improve the therapeutic efficacy in patients with OS.

With the use of the high-throughput sequencing technology, genome databases of patients have been established by researchers for the systematic analysis of the genomic alterations occurring in diseases. Through database mining, researchers have identified many biomarkers and risk signatures to provide new insights into the prognosis of OS [12, 13]. Shi et al. established a metastasis-associated risk signature to predict the survival and metastasis of patients with OS [14]. Also, Hong et al. constructed an immune-related gene signature in OS that could effectively predict the survival of patients [15]. However, to our knowledge, no glycolysis-related risk signature has yet been established till date, for predicting the prognosis of OS. Thus, the establishment of a glycolysis-related gene signature would present a novel strategy for the prognosis and treatment of patients with OS.

Immunotherapy can induce a strong immune response and is a promising treatment method against many tumors [16]. Prior studies have shown the existence of interdependent effects between tumor glycolysis and immune response. For instance, glycolysis contributes to an immunosuppressive effect in non-small cell lung cancer [17]. Wong et al. reported that Staphylococcus aureus small colony variants (SCVs) impair host immunity by activating glycolysis in host cells [18]. However, there is limited research on the relationship between glycolysis and immune response in OS. Thus, there is a need for a more comprehensive understanding of the interaction between glycolysis and the immune response in OS, which would further aid in the development of novel therapeutic strategies for OS.

The aims of our study were to confirm the prognostic glycolysis-related biomarkers of OS and construct a glycolysis-related risk signature, which could accurately predict metastasis and survival rates in patients with OS.

## RESULTS

### Filtering genes through gene set enrichment analysis (GSEA)

**Figure 1:**
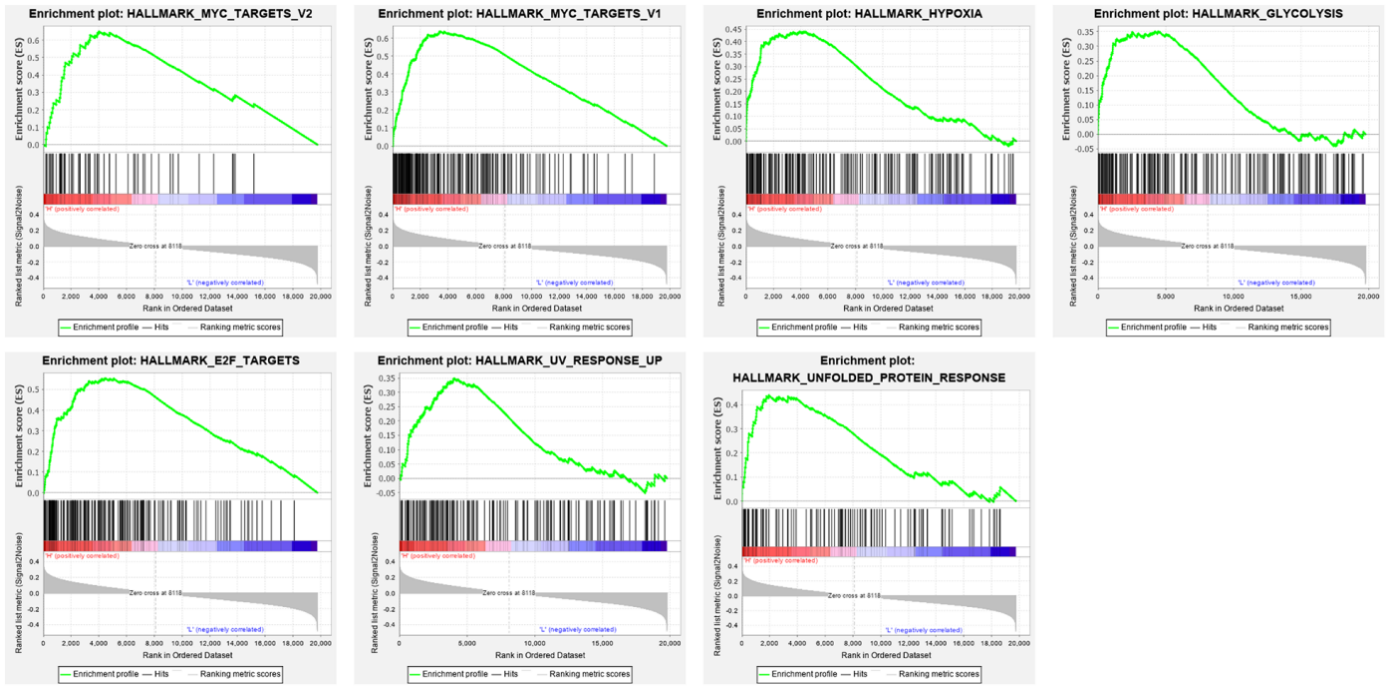
**Enrichment plots of seven gene sets which were importantly differentiated between in metastasis and non-metastasis tissues.** The horizontal axis represents genes in gene sets ranked by decreasing risk score. The vertical axis represents enrichment scores. The enrichment score increased with the number of enriched genes.

The mRNA expression data was downloaded from the genomic data commons data portal (https://portal.gdc.cancer.gov/). The clinical features and prognostic information of the samples were downloaded from the TARGET database (https://ocg.cancer.gov/programs/target). Hallmark gene sets were downloaded from the Molecular Signatures Database (MSigDB). GSEA was performed to analyze specific gene sets among the hallmark gene sets, which differed significantly between metastatic and non-metastatic OS patients. Seven gene sets, namely, UV response, MYC targets V1, MYC targets V2, hypoxia, E2F targets, glycolysis, and unfolded protein response, were significantly enriched (P < 0.05) (Figure 1 and Table 1). As glycolysis has been clearly shown to be related to metastasis [19], and glycolysis in OS is poorly researched, we chose the glycolysis gene set (P = 0.035), which contained 198 genes, to further analyze how the function of glycolysis-related genes differs between patients with metastatic and non-metastatic OS.

**Table 1:** Gene sets enriched in metastasis cancer.

**GS follow link to MSigDB**	**SIZE**	**ES**	**NOM p-value**	**RANK AT MAX**
MYC TARGETS V1	197	0.636776	0.014084507	3370
E2F TARGETS	197	0.55408	0.03522505	4489
HYPOXIA	194	0.440736	0.018595042	3931
UNFOLDED PROTEIN RESPONSE	112	0.438879	0.026422765	1923
MYC TARGETS V2	58	0.649309	0.04	4004
GLYCOLYSIS	198	0.350658	0.034623217	4468
UV RESPONSE UP	157	0.349086	0.020920502	4062

### Identification and construction of a three-mRNA signature to predict the prognosis of patients with OS

**Figure 2:**
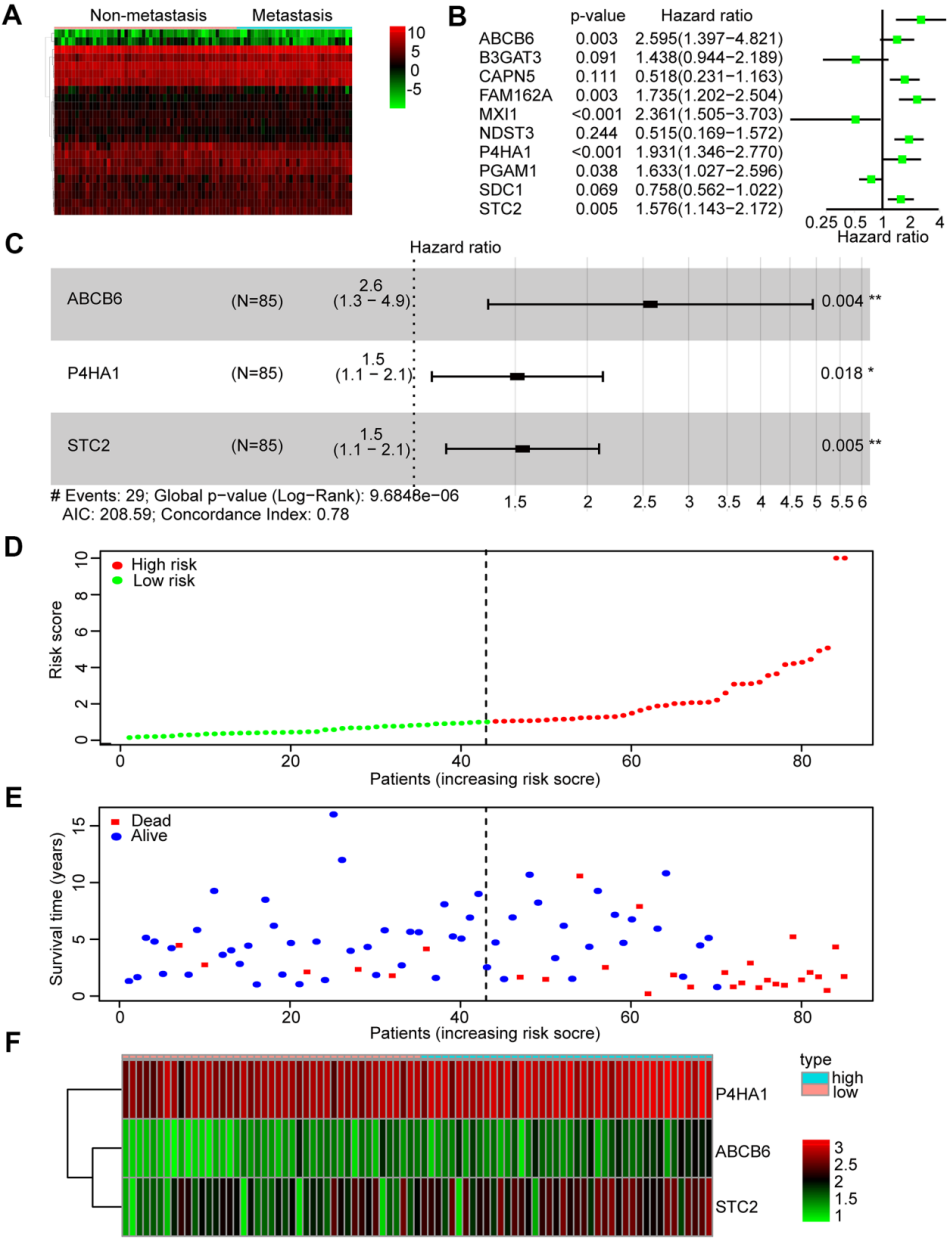
**Identification and construction of a three-mRNA signature to predict the prognosis of patients with OS.** (**A**) Heatmap was constructed showing 23 differentially expressed glycolysis-related genes in metastasis OS tissues compared with non-metastasis tissues. (**B**) Forest plot of univariate Cox regression analysis of the survival-related 10 differentially expressed genes in OS. (**C**) Multivariate cox regression analysis identified a risk signature include 3 glycolysis-related genes among the 10 differentially expressed genes. (**D**) Distribution of risk score in the high-risk group and the low-risk group. (**E**) Survival status between the high-risk group and the low-risk group. (**F**) Heatmap of the expression profile of the included glycolysis-related genes.

According to the glycolysis gene set in MSigDB, 198 glycolysis-related genes were selected. First, we compared the expression profiles of these genes between metastatic and non-metastatic samples in the TARGET database. A heatmap was drawn to illustrate the expression profiles (Figure 2A). We found 23 glycolysis-related genes, including TXN, ABCB6, SLC16A3, CAPN5, B3GAT3, ENO1, PGAM1, ANKZF1, P4HA1, and FAM162A, that were altered in metastatic tissues. Then, we performed Kaplan-Meier (KM) analysis and found that 10 of the 23 genes showed a high correlation with the overall survival rates of patients with OS (Supplementary Figure 1).

Subsequently, we performed a univariate Cox regression analysis of these 10 genes to further search for genes associated with patients’ overall survival and prognosis. Six genes were found to correlate with the prognosis of patients (P < 0.05) (Figure 2B). Finally, these mRNAs were performed by multivariate Cox regression analysis in order to establish a risk signature. Accordingly, three genes (ABCB6, P4HA1, and STC2) were identified as “risk genes” (Figure 2C and Table 2). We calculated the risk scores of all samples in the dataset by applying this three-mRNA signature as follows: expression of ABCB6 × 0.946 + expression of P4HA1 × 0.413 + expression of STC2 × 0.435. After ranking the risk scores, we considered the median score as the cut-off and classified the samples into low- and high-risk groups (Figure 2D). Thereafter, the survival rates of patients in the two groups were compared; low death rates and high survival rates were sorted in the low-risk group (Figure 2E). We also drew a heatmap to illustrate the expression profiles of the three genes in both groups (Figure 2F). The expression of all three genes was low in the low-risk group. Thus, our constructed risk signature could be a good predictor for the prognosis of OS patients.

**Table 2:** Multivariate cox regression analysis identified 3 glycolysis-related genes that are independent factors for OS risks.

**Genes**	**Coefficient**	**HR**	**HR.95L**	**HR.95H**	**P-value**
ABCB6	0.945984	2.575345	1.345086	4.93084	0.00431
P4HA1	0.413458	1.512038	1.073961	2.128809	0.017848
STC2	0.434751	1.544579	1.137423	2.097481	0.005357

### Predicting survival rate and metastasis in OS patients based on the glycolysis-related risk signature

**Figure 3:**
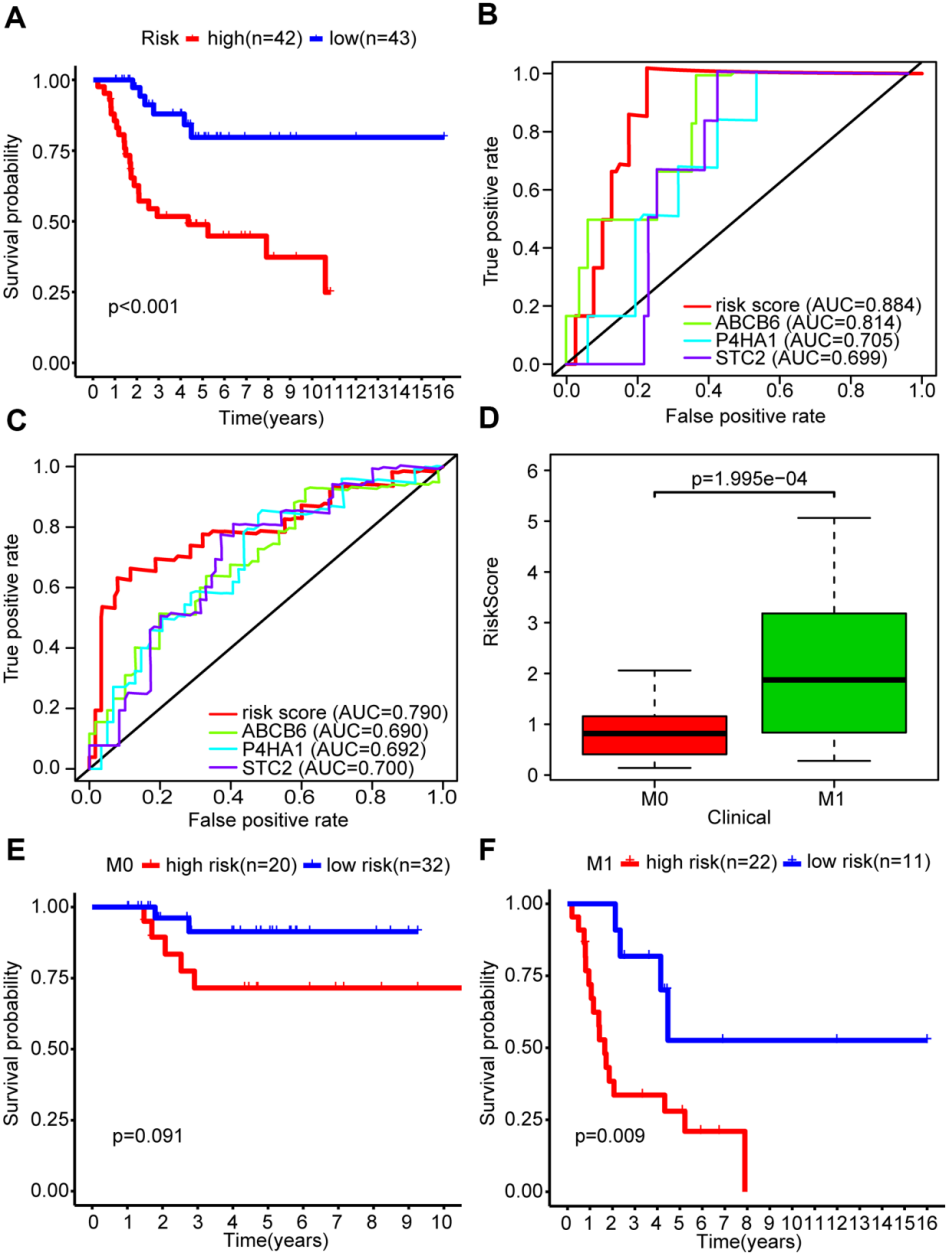
**The glycolysis-related risk signature could predict survival rate and metastasis of OS patients.** (**A**) Kaplan-Meier survival curve of overall survival rate among OS patients from the low-risk group and the high-risk group. Patients in high-risk group had the poorer prognosis. (**B**) Survival prediction between risk score and single gene was assessed by time-dependent receiver operating characteristic (ROC) curve for 1 years. (**C**) Survival prediction between risk score and single gene was assessed by time-dependent receiver operating characteristic (ROC) curve for 3 years. (**D**) Risk scores among metastasis and non-metastasis groups. (**E**, **F**) Kaplan-Meier survival curve of overall survival rate for prognostic value of risk signature for the patients divided by clinical feature of metastasis.

We used Kaplan-Meier analysis to study the differential survival rate between the two groups and observed a significantly higher survival rate was shown in the low-risk group (P < 0.001) (Figure 3A). Next, we performed a receiver operating characteristic (ROC) curve analysis to assess whether the risk signature was efficacious in forecasting outcomes for OS patients. The areas under the curve (AUC) in this risk signature were 0.884 and 0.790 for predicting 1- and 3-year survival, respectively (Figure 3B, 3C). We also used the ROC curve analysis to compare the predictive efficiencies of the three-gene signature and the single genes alone. The results showed that the three-mRNA signature indeed had a higher AUC value for predicting the survival rate of OS patients compared to the individual genes, for both 1 year and 3 years. (Figure 3B, 3C).

The primary cause of death in OS patients is lung metastasis. We found that the metastatic group in our study showed higher risk scores than the non-metastatic group (P < 0.001) (Figure 3D). We stratified OS patients into two subgroups based on their metastasis status and found that the risk signature was more efficient in predicting the overall survival rates of metastatic patients (P < 0.01) compared to non-metastatic patients (P = 0.091) (Figure 3F), indicating that the risk signature was associated with metastasis in OS.

Additionally, we explored whether the risk signature could represent glycolysis activity. Firstly, we performed GSEA and found that four glycolysis-related gene sets (GO_GLYCOLYTIC_PROCESS, HALLMARK_GLYCOLYSIS, KEGG_GLYCOLYSIS_GLUCONEOGENESIS, and REACTOME_GLYCOLYSIS), were significantly enriched in the high-risk group (Supplementary Figure 2A and Table 3). Secondly, core genes in the glycolytic pathway, including HK2, PGAM1, ENO1, and LDHA, showed an elevated expression in the high-risk group (P < 0.001) (Supplementary Figure 2B). These results suggested that the risk signature could simultaneously reflect the activity of glycolysis and predict the survival and metastasis of patients with OS.

**Table 3:** Gene sets enriched in high-risk group in osteosarcoma patients.

**GS follow link to MSigDB**	**SIZE**	**ES**	**NOM p-value**	**RANK AT MAX**
HALLMARK_GLYCOLYSIS	198	0.38	0.011	3597
KEGG_GLYCOLYSIS_GLUCONEOGENESIS	62	0.49	0.006	4139
GO_GLYCOLYTIC_PROCESS	104	0.43	0.012	2731
REACTOME_GLYCOLYSIS	72	0.45	0.035	370

### Independence of the three-mRNA signature in predicting the prognosis of patients with osteosarcoma

**Figure 4:**
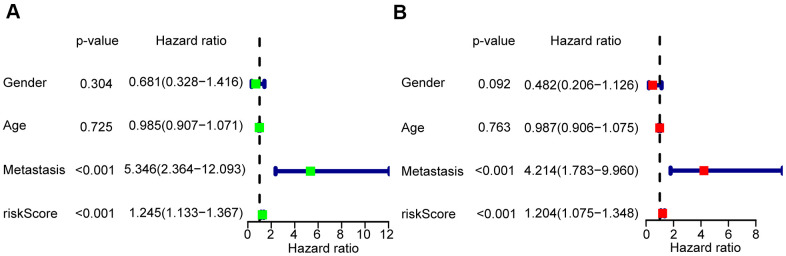
**Risk score based on the glycolysis-related risk signature could be an independent prognostic factor.** (**A**) A forest plot of univariate cox regression analysis of risk score and different clinical feature in OS. (**B**) A forest plot of multivariate cox regression analysis of risk score and different clinical feature in OS.

To verify whether the risk scores were independent of other clinical characteristics, we performed univariate and multivariate Cox regression analyses. The age of 15 was the median age among the 85 OS patients, with 48 male and 37 female patients were included in the TARGET database. This group included 33 patients who presented with metastasis, and 52 without. Through both univariate and multivariate Cox analyses, the risk scores and the clinicopathological parameters of metastasis were demonstrated to be independent prognostic factors in OS to predict the prognosis of OS patients (P < 0.001) (Figure 4A, 4B).

### Validation of the prognostic risk signature

**Figure 5:**
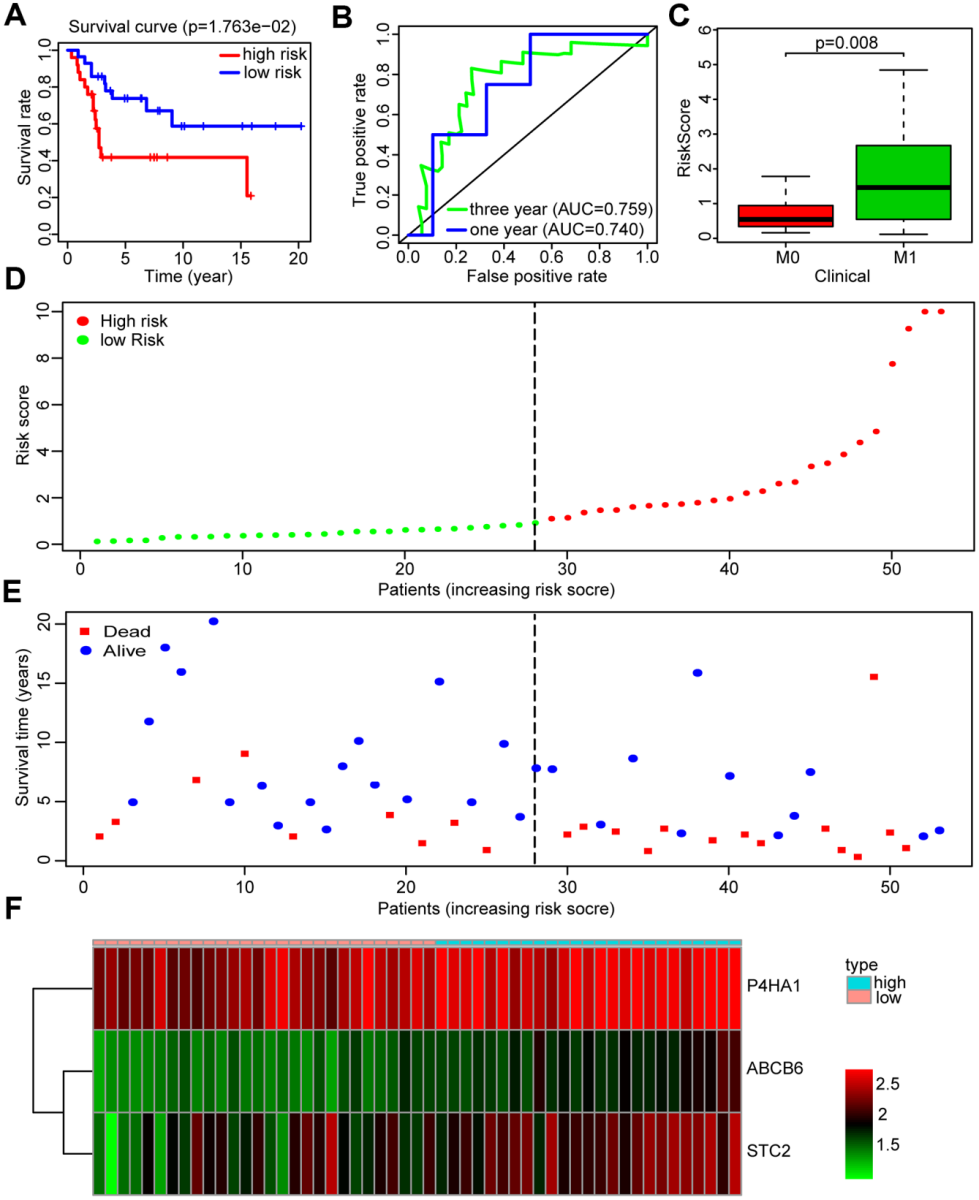
**Validation of the prognosis risk signature in GSE21257.** (**A**) Kaplan-Meier survival curve of overall survival rate among OS patients from the low-risk group and the high-risk group in GSE21257. Patients in high-risk group had the poorer prognosis. (**B**) Survival prediction of the risk signature in GSE21257 was assessed by time-dependent receiver operating characteristic (ROC) curve for 1 and 3 years. (**C**) Risk scores among metastasis and non-metastasis groups in GSE21257. (**D**) Distribution of risk score in the high-risk group and the low-risk group in GSE21257. (**E**) Survival status between the high-risk group and the low-risk group in GSE21257. (**F**) Heatmap of the expression profile of the included glycolysis-related genes in GSE21257.

A GEO data set (GSE21257) was chosen by us as a validation cohort to ensure the reliability of the established glycolysis-related risk signature. This cohort comprised 53 cases of which 25 were in the high-risk group and 28 in the low-risk group, based on the risk signature. Low survival rates of patients were consistently seen in the high-risk group (Figure 5A). The risk signature’s prognostic efficiency was assessed using the ROC curve analysis in the validation group; the AUC was 0.740 at 1 year and 0.759 at 3 years (Figure 5B). Moreover, in this validation cohort patients with metastasis had a higher risk score than those without metastasis (P<0.01) (Figure 5C). Higher mortality and lower patient survival rates were observed in the high-risk group compared with the low-risk group (Figure 5D, 5E). A heatmap was drawn according to the expression profiles of the risk genes between both groups (Figure 5F). We found that the risk genes showed a higher expression in the high-risk group compared to the low-risk group. We further verified the prognostic potential of the three glycolysis-related risk genes in the R2 database (http://r2.amc.nl). The high-P4HA1 expression group had a lower overall survival probability and metastasis-free survival probability than the low-P4HA1 expression group (Supplementary Figure 3A). The high-STC2 expression group had a lower overall survival probability than the low-STC2 expression group (Supplementary Figure 3B). The high-ABCB6 expression group also showed a lower metastasis-free survival probability than the low-ABCB6 expression group (Supplementary Figure 3C). We also collected five metastatic human OS tissues and five non-metastatic human OS tissues. The results of western blotting showed that the risk genes (P4HA1, ABCB6, and STC2) had higher expressions in metastatic human OS tissues than non-metastatic human OS tissues (Supplementary Figure 4). These results demonstrated that the risk model constructed from the three risk genes was a good predictive instrument for prognosis in the validation cohort.

### Changes in the tumor microenvironment of OS patients based on the glycolysis-related risk signature

**Figure 6:**
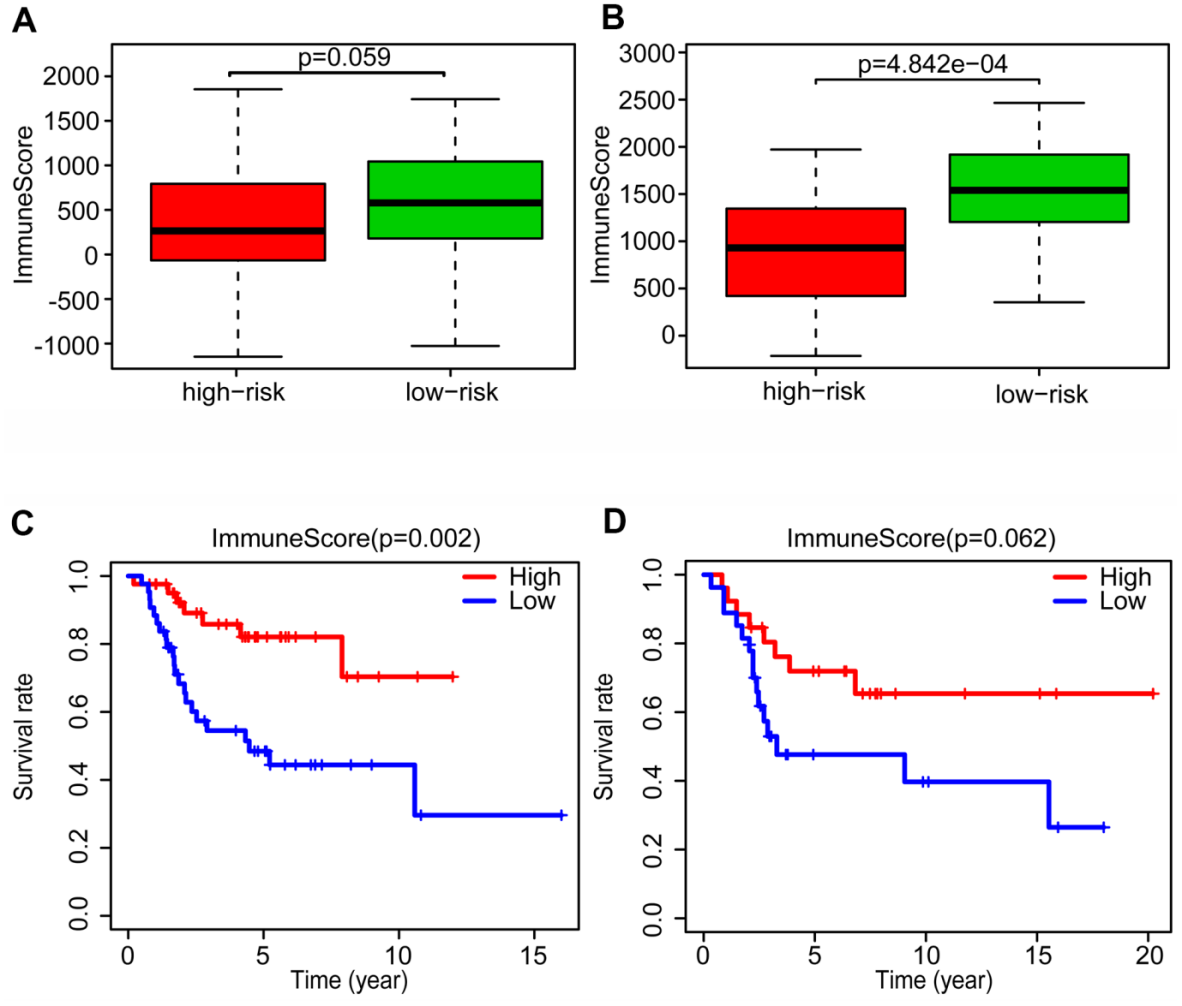
**Changes of tumor microenvironment of osteosarcoma patients based on glycolysis-related risk signature.** Immune score was calculated between high-risk and low-risk group in training cohort (**A**) and validation cohort (**B**). Kaplan-Meier survival curve of overall survival rate among high-risk and low-risk group in training cohort (**C**) and validation cohort (**D**).

Recent research has emphasized the pivotal importance of whole organism nourishment and metabolism in the function of immune cells [31], but the relationship between immune response and glycolysis in OS is unknown. To further explore the potential connection between immune response and glycolysis in OS, we calculated the immune score of the patients in the training and validation cohorts, using the ESTIMATE algorithm. In the training cohort, we found that the patients in the low-risk group had immune scores higher than those in the high-risk group (P = 0.059, almost statistically significant) (Figure 6A), and the similar results were seen in the validation cohort (P < 0.001) (Figure 6B).

**Figure 7:**
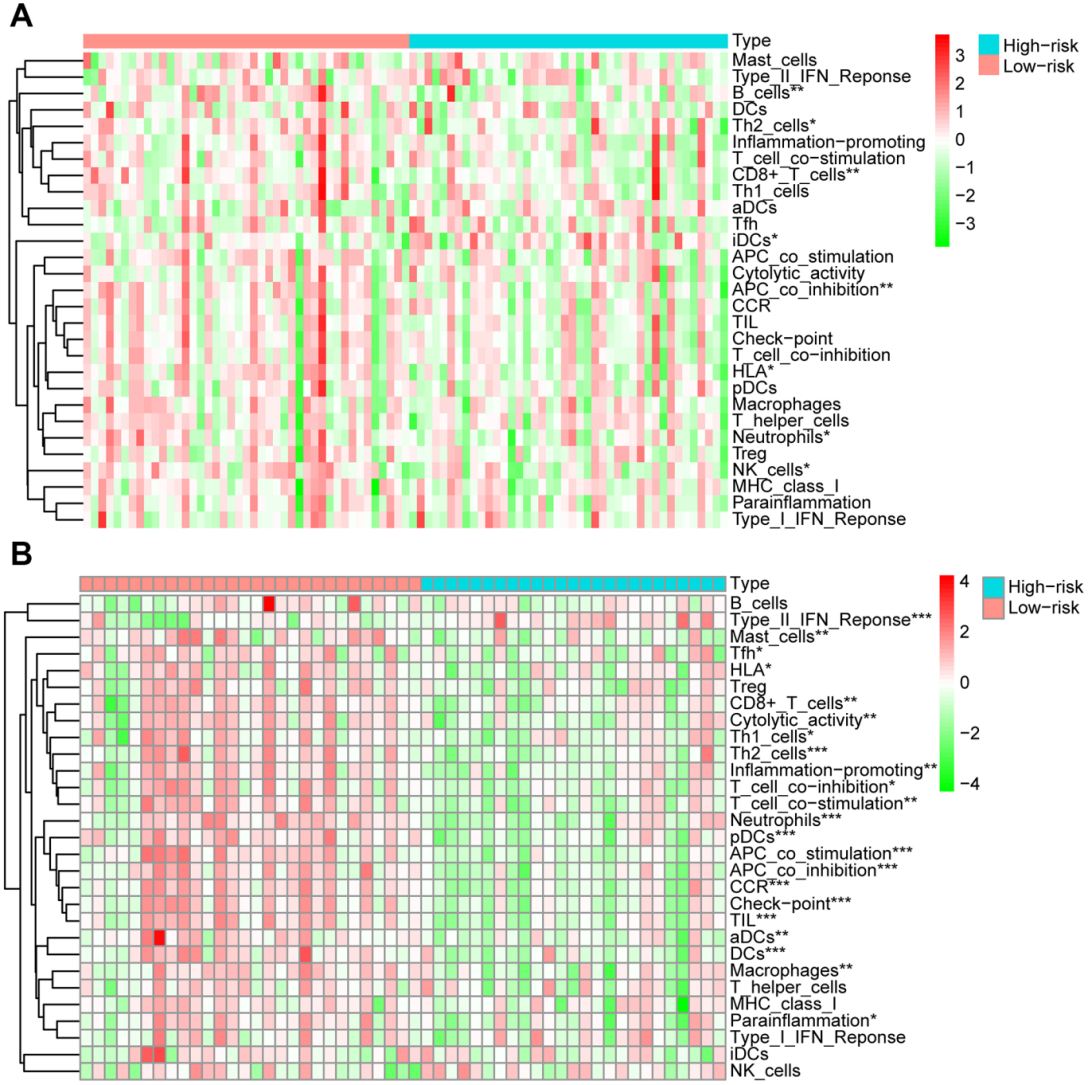
**Analysis of 29 immune gene sets between high- and low-risk groups of OS.** The heatmap was used to visualize the proportions of these gene sets between high- and low-risk groups of OS in training cohort (**A**) and validation cohort (**B**).

**Figure 8:**
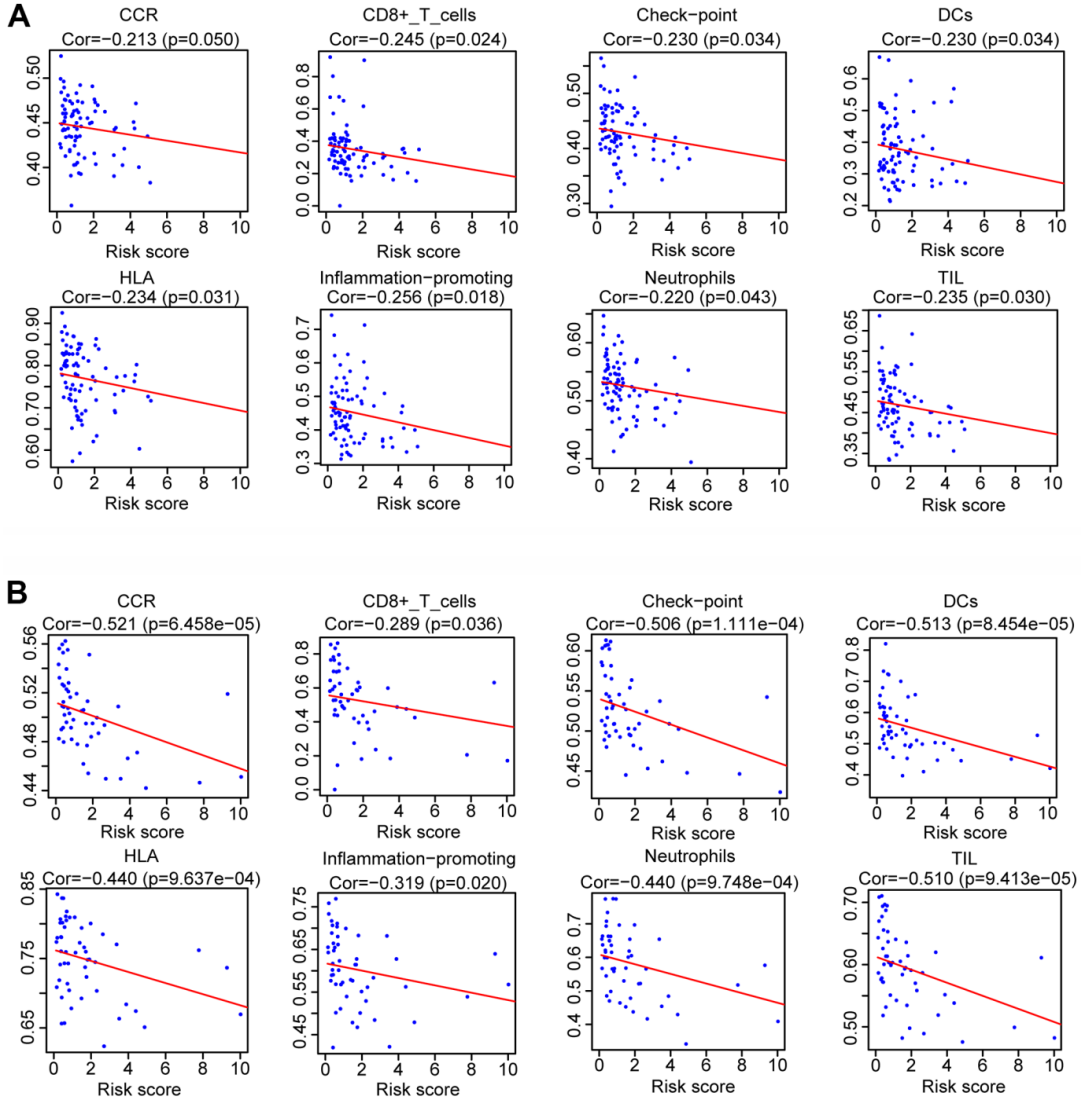
**Analysis of the correlation between risk scores and the immune gene sets.** Spearman rank analysis was used to determine the association between risk score and the immune gene sets in training cohort (**A**) and validation cohort (**B**).

After dividing the patients into high- and low-immune score groups according to the median value of the immune scores in the training and validation cohorts, we performed Kaplan-Meier survival analysis of the data. The results showed that a higher immune score was related to a good prognosis in both the training cohort (P < 0.01) (Figure 6C) and validation cohorts (P = 0.062, almost statistically significant) (Figure 6D). In addition, we performed single-sample gene set enrichment analysis (ssGSEA) to calculate the relative abundance of 29 immune-related items of each sample in both cohorts. The 29 immune gene sets included immune cell types and functions, tumor-infiltrating lymphocytes (TILs), proinflammatory, para-inflammation (PI), cytokine and cytokine receptor (CCR), human leukocyte antigen (HLA), regulatory T (Treg) cells, and immune checkpoint. We found that immune infiltration levels were higher in the low-risk group in both the training cohort (Figure 7A) and validation cohorts (Figure 7B). In particular, the “APC_co_inhibition”, “Neutrophils”, “Th2_cells”, “CD8+_T_cells” and “HLA” gene sets showed differential expression between low- and high-risk groups in both cohorts. Meanwhile, we analyzed the correlation among immune cell types, immune pathways, and the risk scores, and found that the proportions of CCR, CD8+_T_cells, neutrophils, TILs, dendritic cells (DCs), HLA, inflammatory promoting cells and immune checkpoints showed negative correlation with the risk scores in both training cohort (Figure 8A) and validation cohort (Figure 8B). We also verified the differential expression of CD8+ T cell between metastatic and non-metastatic human OS tissues in clinical samples. The result suggested that patients with metastasis had lower contents of CD8+ T cell than those without metastasis (Supplementary Figure 5). Thus, we demonstrated that the risk signature correlated with the immune response and immune infiltration levels in OS samples. Furthermore, these results also showed that high immune infiltration levels could suggest good prognosis in OS patients.

### Validation of the function of P4HA1 *in vitro* and *in vivo*

**Figure 9:**
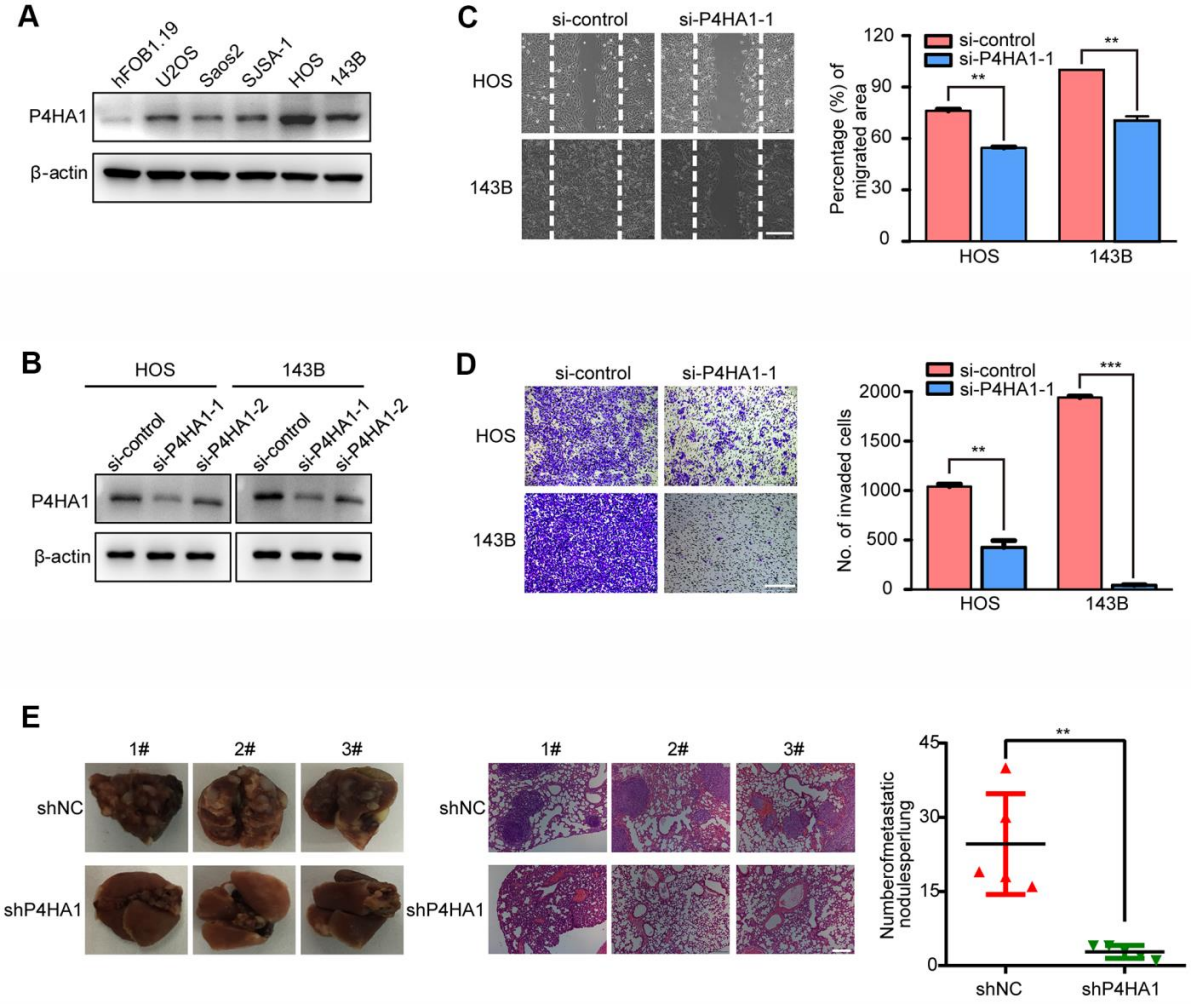
**P4HA1 promoted the metastasis of OS *in vitro*.** (**A**) Western blotting analysis of the expression of P4HA1 in human osteoblastic cell line and osteosarcoma cell lines. (**B**) Western blotting analysis of the expression of P4HA1 in HOS and 143B cell lines after transfection of si-control, si-P4HA1-1 and si-P4HA1-2. (**C**) Wound healing assay analysis in HOS and 143B cell lines after transfection of si-control and si-P4HA1-1. Representative images of migration were shown in the right panel. The degrees to which the wounds healed was shown in the histogram. (**D**) Transwell invasion assay analysis in HOS and 143B cell lines after transfection of si-control and si-P4HA1-1. Representative images of invasion were shown in the right panel. The proportions of invading cells were shown in the histogram. The bars indicate the mean±s.d. Statistically significant differences (t-test), **P<0.01, ***P<0.001. (**E**) The number of lung metastasis nodules formed by P4HA1-knockdown 143B cells and control cells in orthotopic osteosarcoma implanted mice was shown in the right panel. Statistically significant differences (t-test), **P<0.01. Representative images of lung morphology and lung metastatic nodules formed by P4HA1-knockdown 143B cells and control cells were shown in the left panel. Corresponding HE staining was shown in the middle panel. Scale bars = 200 μm.

To further examine the role of the three genes in the metastasis of OS, P4HA1 was analyzed in more detail. We verified the expression of P4HA1 in OS cell lines and the hFOB1.19 human osteoblast. The results of western blotting indicated that P4HA1 showed a higher expression in human OS cells than in the osteoblast cell line hFOB1.19 (Figure 9A). To further verify the function of P4HA1 in OS progression, we transfected si-P4HA1 into HOS and 143B OS cell lines. Knockdown efficiency was verified by western blotting (Figure 9B) and si-P4HA1-1 selected for the subsequent experiments. According to the results of the wound-healing assay, knockdown of P4HA1 could restrain the migration of OS in the HOS and 143B cell lines (Figure 9C). Furthermore, the transwell invasion assay results corroborated with those of the wound-healing assay in HOS and 143B cell lines (Figure 9D), indicating that P4HA1 indeed played a vital role in the metastasis of OS *in vitro*. Next, we further verified the role of P4HA1 in OS metastasis *in vivo*. The numbers of lung metastatic nodules in P4HA1-knockdown orthotopic OS-implanted mice were fewer than those in the control group (Figure 9E). These results further confirmed our previous results (Figure 2A–2C).

## DISCUSSION

Recently, the study of energy metabolism has attracted increasing attention. The Warburg effect can facilitate the growth of cancer cells by promoting biosynthetic pathways of carbon fluxes and the adaptation of cells to hypoxic conditions [20, 21]. OS is a malignant tumor primarily occurring in adolescents and young adults, for which no effective treatment methods or targets are currently available. Therefore, a need exists for research toward the discovery of glycolysis-associated genes that could predict the prognosis of patients with OS.

Although the clinical significance of glycolysis-associated gene signatures in predicting the prognosis of patients with tumors has been demonstrated in many cancers, an analysis on a genome-wide scale for determining the underlying mechanisms of the glycolysis-associated gene signatures in OS is lacking. In endometrial cancer (EC), Wang et al. reported a nine-glycolysis-associated gene signature that can predict the survival of EC patients [22]. Zhang et al. established a glycolysis-related risk signature to predict the prognosis of lung adenocarcinoma patients [23]. In our study, we established a glycolysis- associated risk signature that can serve as independent prognostic factor in predicting the prognosis of OS patients.

Tumor metastasis is the major cause of mortality in OS patients. An increasing number of researchers have been exploring the underlying mechanisms of OS metastasis [24]. Liu et al. identified metastasis-related genes as potential biomarkers of OS via the bioinformatic analysis of the GEO database [25]. Su et al. also reported the metastasis-associated gene MAPK15 through the GEO database that could promote OS metastasis [26]. Here, we first found that the glycolysis-related gene set was enriched in the metastasis group of OS patients using the TARGET database. Thereafter, we selected glycolysis-related differentially expressed genes in metastasis and non-metastasis samples and further constructed a glycolysis-related risk signature to predict the survival and metastasis of OS patients through univariate and multivariate Cox regression analyses. Three genes were ensured in this risk signature (P4HA1, STC2, and ABCB6). P4HA1 encodes the active catalytic component of prolyl 4-hydroxylase.was one of the genes encoding the active catalytic component of prolyl 4-hydroxylase. Aberrant P4HA1 expression affects tumor progression in several malignant tumors [27, 28]. In pancreatic cancer, P4HA1 can promote glycolytic and malignant phenotypes through the P4HA1/HIF1α feedback loop [29] and in ovarian cancer, P4HA1 promotes tumor metastasis by regulating the epithelial-mesenchymal transition (EMT) [30]. Likewise, we verified the expression of P4HA1 in OS cell lines and found that P4HA1 showed higher expression levels in OS cells than in hFOB1.19 osteoblasts. In the wound-healing migration assay and transwell invasion assays, we found that the knock-down of P4HA1 restrained the metastasis of OS cell lines. Our results demonstrate that P4HA1 is associated with poor prognosis and can promote metastasis of OS *in vitro* and *in vivo*. ABCB6, an ATP-binding cassette (ABC) transporter group member, is one of the hallmark genes in glycolysis. There are many biological effects of ABCB6, such as protection against oxidative stress [31], acquired resistance [32, 33], and the potential promotion of tumor proliferation [34, 35]. Consistently, our results showed that ABCB6 was overexpressed in patients with metastatic OS and a high expression of ABCB6 indicated a low survival rate in OS patients. STC2, a human ortholog of fish stanniocalcin, is widely expressed in many tissues. STC2 shows significant upregulation in pancreatic cancer tissues and contributes to metastasis by promoting EMT in pancreatic cancer [36]. In head and neck squamous cell carcinoma, STC2 is regulated by microRNA-206 and influences cell proliferation and invasion [37]. In our study, we showed that STC2 also contributed to the progression of OS and was associated with poor prognosis of OS patients.

Cancer metastasis to distant organs is the dissemination of primary heterogeneous tumors. Different metabolic changes, such as glycolysis, contribute to the survival of tumor metastases. Feng et al. illustrated that aerobic glycolysis could promote the growth and metastasis of hepatocellular carcinoma [38]. In pancreatic cancer, Nie et al. found that ALDH1A3 promoted the metastasis of tumor cells by enhancing the glycolysis in them [39]. In our study, we divided the patients into high- and low-risk groups according to an established risk signature. According to the GSEA results, the “GO_GLYCOLYTIC_PROCESS,” “HALLMARK_GLYCOLYSIS,” “KEGG_GLYCOLYSIS_GLUCONEOGENESIS,” and “REACTOME_GLYCOLYSIS” gene sets were markedly enriched in the high-risk group. Moreover, the key genes of the glycolytic pathway, including HK2, PGAM1, ENO1 and LDHA, showed higher levels of expression in the high-risk group, suggesting that the three-mRNA signature represented the glycolysis level in OS patients. As the risk signature accurately predicted the prognosis of OS patients in training cohorts and validation cohorts, we deduced that a high glycolytic activity was related to a poor prognosis and high potentiality of metastasis in OS patients.

The relationship between glycolysis and immune response has been reported by many researchers. Souto-Carneiro et al. found that increased glycolytic activity promotes the proinflammatory profile of CD8+ T cells [40]. Also, Hu et al. showed that overexpression of CD47 plays an important part in tumor cell immune evasion and enhances glycolysis in colorectal cancer cells [41]. Likewise, we used the ESTIMATE algorithms and ssGSEA to analyze the OS samples in the two cohorts and discovered that patients in the high-risk group had lower immune scores and immune infiltration levels than those of the low-risk group. Petitprez et al. revealed that B cells played a critical role in the immunotherapy response of patients with sarcoma and were associated with the prognosis of patients [42]. Milcah et al. found that decreased immune cells correlated with metastasis and poor prognosis of patients with OS [43]. Consistent with these findings, we revealed that the risk score calculated from our established risk signature was negatively correlated with the proportions of CCR, CD8+_T_cells, neutrophils, TILs, dendritic cells (DCs), HLA and inflammatory promoting cells in both training and validation cohorts. Therefore, we suggested that immune infiltration levels of OS were correlated with the glycolysis-related risk signature and the underlying mechanisms need to be further explored.

Although the risk signature of three genes was emphasized by us in predicting the prognosis of patients with OS, and the function of P4HA1 was verified in OS cell lines and *in vivo*, some limitations still existed and needed further inquiry. First, because the sizes of the OS samples in our training and validation cohorts were small, further verification is needed with the use of larger cohorts. Second, apart from P4HA1, the function of the other two genes contained in our risk signature should also be verified *in vitro*.

In this study, we identified, for the first time a three-gene risk signature related to glycolysis that could predict the prognosis of OS patients. Furthermore, we also found that the risk signature was associated with the immune infiltration levels of OS. These findings may not only provide a new tool to predict the prognosis of OS patients but also offer novel therapeutic targets for the treatment of OS patients.

## MATERIALS AND METHODS

### Data gathering

We downloaded the mRNA expression data of patients with OS from the genomic data commons data portal (https://portal.gdc.cancer.gov/). Complete clinical information of the above patients were downloaded from the TARGET database (https://ocg.cancer.gov/programs/target). The mRNA expression data and clinical characteristics of OS samples in GSE21257 were downloaded from the NCBI gene expression omnibus (https://www.ncbi.nlm.nih.gov/geo/, GSE21257). All of the clinical information in the two datasets were shown in Supplementary Table 1.

### Gene set enrichment analysis (GSEA)

Gene sets which were significantly different between non-metastasis and metastasis groups in OS samples were screened out by performing GSEA (http://www.broadinstitute.org/gsea/index.jsp). The glycolysis-related genes were downloaded from the Molecular Signatures Database (MSigDB) in GSEA.

### Establishment and validation of a glycolysis-related risk signature

We performed univariate cox analysis and Kaplan-Meier (KM) survival analysis to assess the role of glycolysis-related genes played in predicting the prognosis of OS patients. Statistically different genes acquired in both univariate variable Cox analysis and KM survival analysis were selected for further analysis in multivariable Cox analysis. Then, we obtained the prognostic risk score formula through the multivariate cox regression analysis according to a linear combination of expression levels weighted with the regression coefficients. The formula was as follows:

$$\text{Risk score=}{\sum^{\text{​}}}_{i=1}^{n}({\text{coef}}_{\text{i}}*{\text{Expr}}_{\text{i}})$$

where Expr_i_ is the expression levels of gene i in a particular patient and coef_i_ is the coefficients of the gene i in the multivariate cox regression analysis. The median of risk scores were took by us as the cut-off, through which the patients were divided into high- and low-risk groups. Next, the KM survival analysis was performed to assess the differential survival rate between different groups and the ROC curves were used to evaluate the predictive efficiency of this risk signature. At last, we performed univariate and multivariate cox regression analyses to verify whether the risk score calculated by risk signature could be an independent prognostic factors of OS patients in the TARGET dataset. To further verify the accuracy of the risk signature, we chose the GSE21257 dataset as the validation cohort containing 53 patients with OS, which had intact clinical information. All of data in the two cohorts were adjusted in R software via the “sva” package. The TARGET dataset was recognized as the training cohort. According the risk signature, patients in GSE21257 were divided into low-and high-risk groups. Lastly, we performed KM survival analysis and ROC curves to assess the efficiency and accuracy of the risk signature in GSE21257 dataset.

### Immune scores in OS samples

Firstly, we used the “limma” package in R software to normalize the matrix data of gene expression [44]. Then, immune score of each sample was computed by ESTIMATE algorithm according to the normalized gene expression [45].

### SsGSEA in OS samples

To quantify the proportions of immune signatures in the OS samples based on the ssGSEA score, we used the 29 immune signatures, including cell types, functions, and pathways [46].

### Immunohistochemistry (IHC)

IHC assays were conducted as reported previously [47]. Images were obtained with Leica microscope (Leica, DM4000b).

### Osteosarcoma cell lines

The OS cell lines (143B, U2OS, Saos2, HOS and SJSA-1) and osteoblast cell line (hFOB1.19) were acquired from ATCC (Manassas, VA, USA). DMEM culture medium mixed with 10% FBS and 1% penicillin/streptomycin was used to culture the cell lines. All cell lines were cultivated in the incubator at 37° C with 5% CO2.

### Western blotting

Firstly, we used the RIPA buffer (Beyotime, Shanghai, China) mixed with a protease and a phosphatase inhibitor cocktail (Sigma-Aldrich, USA) in advance to lyse the cells for 30 minutes on ice. After centrifugation, supernatant of lysate was mixed with loading buffer (Beyotime, Shanghai, China). After degeneration of lysates, they were separated by SDS-PAGE. The Bio-Rad (Hercules, CA, USA) was used shift the protein from SDS-PAGE to polyvinylidene difluoride (PVDF) membranes. Then, 5% non-fat milk diluted by TBST was incubated with the PVDF membranes for one hour at room temperature, after which the membranes were incubated with primary antibodies at 4° C overnight. The next day, TBST was used to wash the membranes for three times after which incubated with secondary antibody (Sigma-Aldrich) for 1h at room temperature. Lastly, after washing the membranes for three times by TBST, ECL detection reagents were used to detect the signals. Primary antibodies: P4HA1 (12658-1-AP, Proteintech).

### RNA interference

P4HA1 siRNA duplexes and corresponding si-Control were purchased from RiboBio (Guangzhou, China). Lipofectamine 3000 reagent (Invitrogen, Carlsbad, CA, USA) was used to transfect cells based on the manufacturer's protocol. The sequences targeting P4HA1 were as follows: 5’-CCTGTGGTGTCTCGAATTAATdTdT-3’ (si-P4HA1-1); 5’- GGAAUUACAGGUAGCAAAU-3’(si-P4HA1-2).

### Transwell invasion assay and wound-healing migration assay

Cell invasion capabilities were detected by using Transwell chambers precoated with the Matrigel matrix as previously described [47]. We seeded 5*10^4^ cells into the upper compartment with 100μl serum-free culture medium. Meanwhile, we added 600μl DMEM with 10% FBS into the lower compartment as a chemoattractant. Then, the plates were incubated for 12h at 37° C, after which we used a cotton bud to clear the non-invaded cells in the upper chamber and used 4% paraformaldehyde to fix the invaded cells on the bottom surface. Fifteen minutes later, 0.1% crystal violet was used to stain the invaded cells. We used an inverted microscope (Olympus) to acquire the images, and the corresponding cells were counted by ImageJ software (National Institutes of Health, MD). Then, we performed the wound-healing migration assay. Six-well plate was used to seed the cells at the density of 80% in advance. Then, we used a sterile 100 μL pipette tip to create a “wound” when the cells reached full confluence. Afterwards, we observed the breadth of “wound” at different time points with a fluorescence microscope (Olympus). The distances were measured by ImageJ software (National Institutes of Health, MD).

### Cell transfection

The P4HA1 lentiviral shRNA plasmid was supplied by Genomeditech (Shanghai, China). The sequences targeting P4HA1 was as follows: shP4HA1, CCGGCCTGTGGTGTCTCGAATTAATCTCGAGATTAATTCGAGACACCACAGGTTTTTG. The lentivirus was attained by transferring the plasmid into HEK-293T cell lines with Lipofectamine 3000 (Invitrogen, Carlsbad, CA, USA) in according with the manufacturer’s instructions.

### *In vivo* animal experiments

The animal experiments were approved by the Animal Research Committee of the Shanghai General Hospital (Ethical code: 2019AW004) and were performed in accordance with established guidelines. For the growth and metastasis assays *in vivo*, four-week-old male nude mice were orthotopically inoculated into the right tibia with a micro syringe. A total of 1 × 10^6^ corresponding cells suspended in 20 μL of PBS were injected into each nude mouse. One month later, the mice were sacrificed, the tumors and individual lung tissues were excised and fixed with 4% paraformaldehyde. Metastatic lung tissues were analyzed by H&E staining.

### Statistical analysis

Statistical analyses were performed mostly based on the R 3.5.1 (https://www.r-project.org/) and GraphPad Prism 5 (GraphPad Software Inc, La Jolla, CA). The univariate cox regression analysis was used to assess the correlation between gene expressions and prognosis of patients. The multivariate cox regression analysis was used to establish the risk signature based on the genes related to prognosis of patients with OS. Differential analysis of survival rates between high- and low-risk groups generated by the KM analysis was defined by log-rank tests. The package of “survival ROC” in R software was utilized to generate ROC curve to assess the predictive ability of the established risk signature. P < 0.05 was considered be significant in all statistical tests.

## Supplementary Material

Supplementary Figures

Supplementary Table 1
