# Supplementary material for: Identification of a novel glycolysis-related gene signature for predicting the prognosis of osteosarcoma patients
Source: Aging (Albany NY). 2021 May 5;13(9):12896–918. doi: 10.18632/aging.202958 (PMC8148463; doi:10.18632/aging.202958)
Supplement: Supplementary Figures [file aging-13-202958-s001.pdf]

SUPPLEMENTARY FIGURES

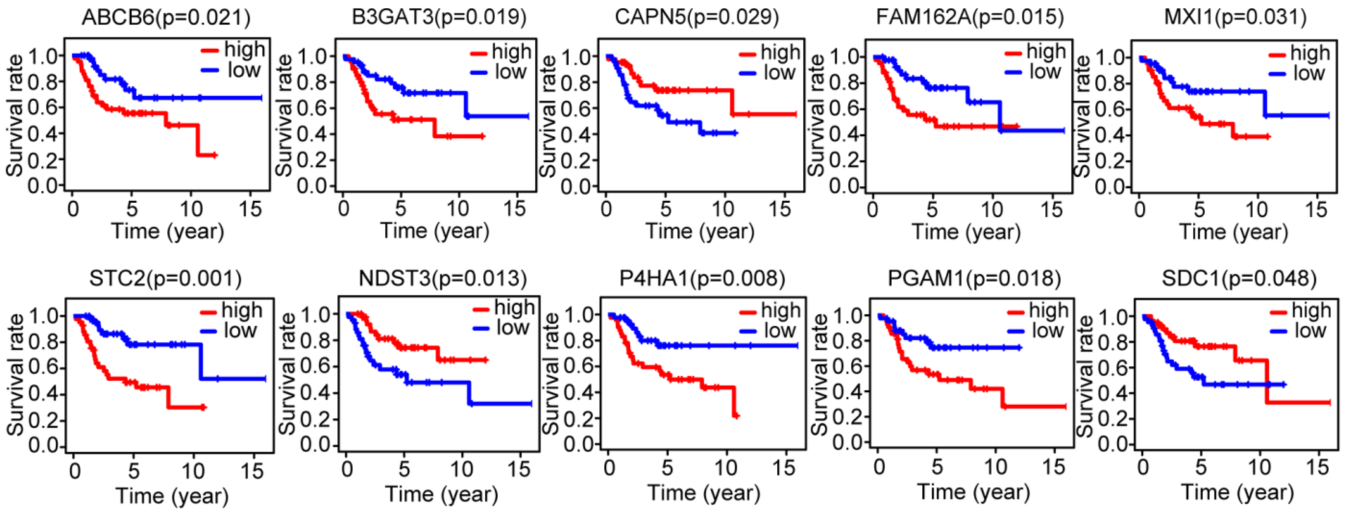

Supplementary Figure 1. Kaplan-Meier survival curve analysis of the ten genes related to overall survival rate.

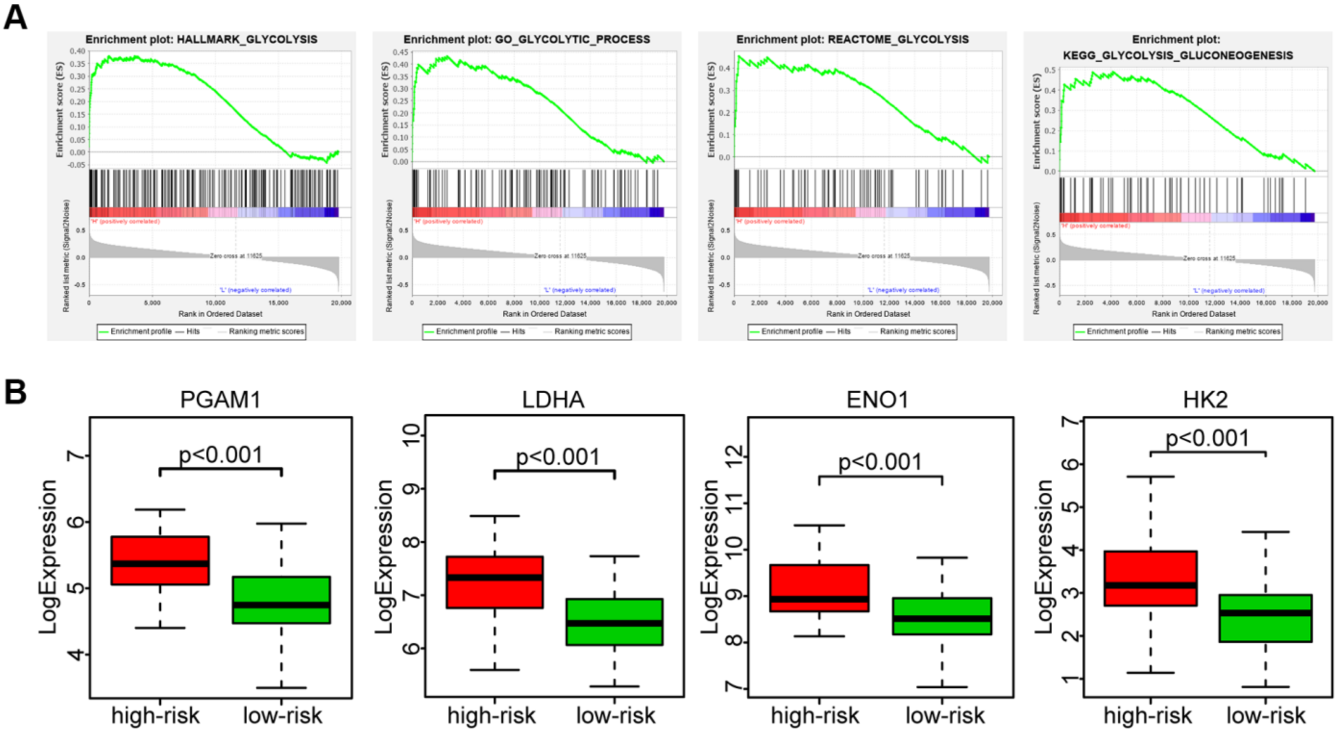

Supplementary Figure 2. The risk signature can reflect the activity of glycolysis to some extent. (**A**) Enrichment plots of four glycolysis-related gene sets which had significant difference between high-risk and low-risk groups by performing GSEA. (**B**) The expression of glycolytic pathway-specific genes were higher expressed in high-risk group than low-risk group.

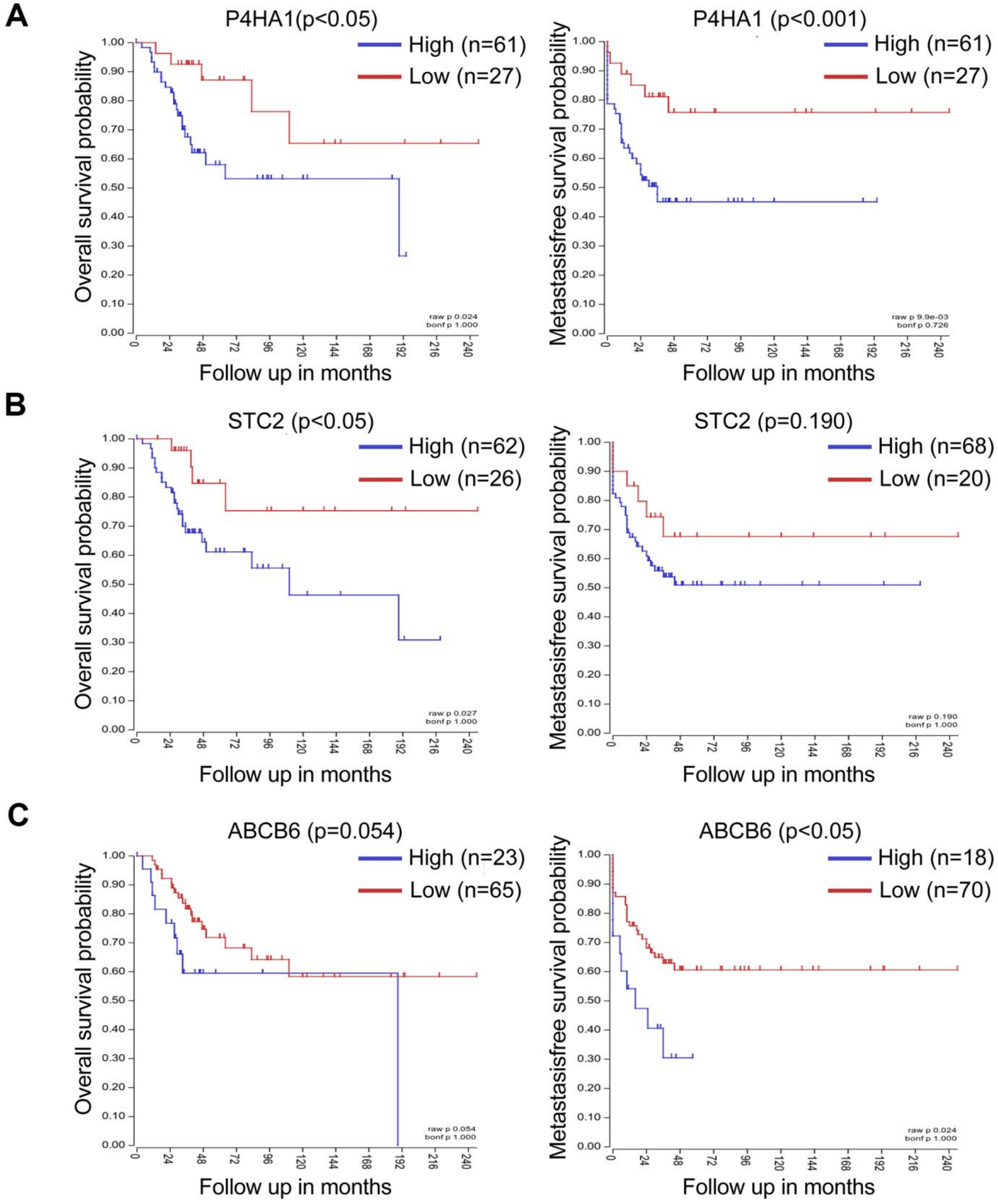

Supplementary Figure 3. Validation of the risk genes in R2 database. (**A**) Kaplan-Meier survival curve of overall survival rate and metastasis free survival rate among OS patients from low-P4HA1 expression group and high-P4HA1 expression group. (**B**) Kaplan-Meier survival curve of overall survival rate and metastasis free survival rate among OS patients from low-STC2 expression group and high-STC2 expression group. (**C**) Kaplan-Meier survival curve of overall survival rate and metastasis free survival rate among OS patients from low-ABCB6 expression group and high-ABCB6 expression group.

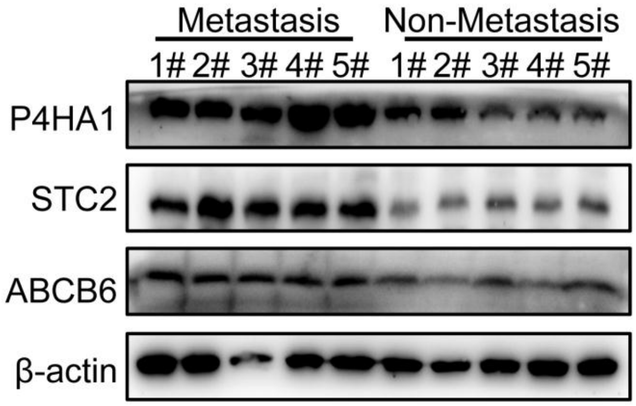

Supplementary Figure 4. The risk genes are upregulated in metastatic human OS tissues. Western blot analysis of risk genes' expression in metastatic human OS compared with non-metastatic human OS tissues.

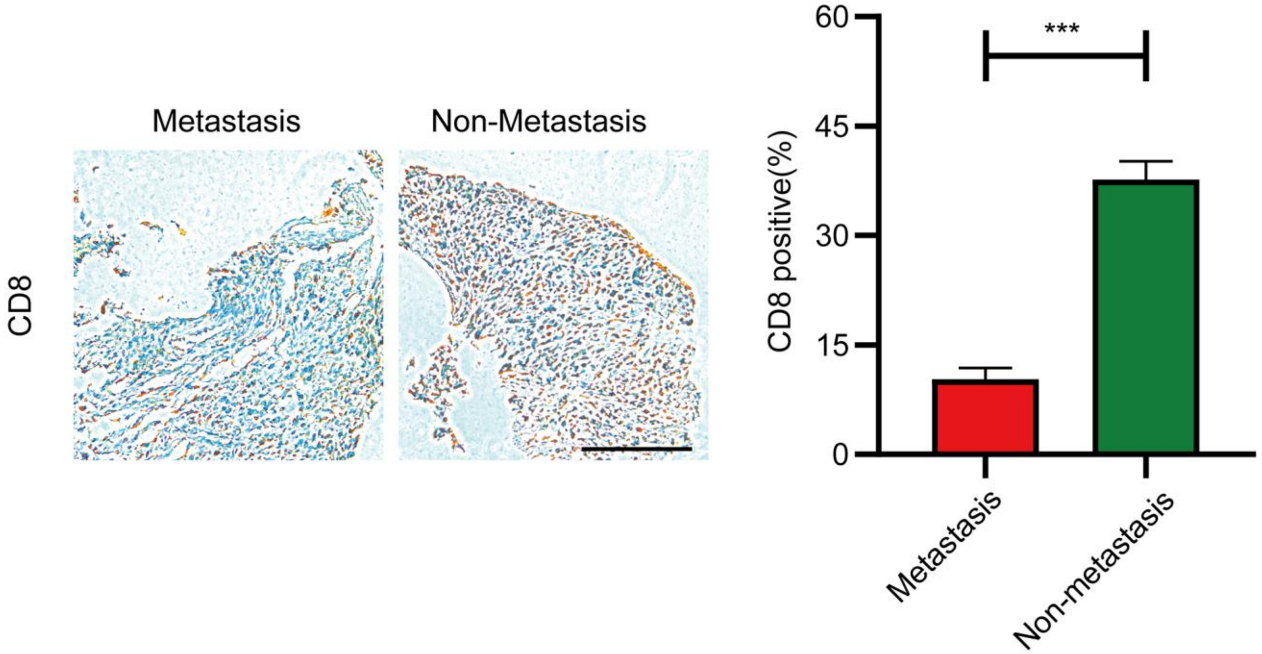

Supplementary Figure 5. The expression of CD8+ T cell was detected by IHC in metastatic and non-metastatic human OS tissues. The results of CD8+ T cell positive (%) were shown in the right panel. The bars indicate the mean±s.d. Statistically significant differences (t-test), ***P<0.001, Scale bars = 250 μm.
