# Supplementary material for: Identification of a novel glycolysis-related gene signature for predicting the prognosis of osteosarcoma patients
Source: Aging (Albany NY). 2021 May 5;13(9):12896–918. doi: 10.18632/aging.202958 (PMC8148463; doi:10.18632/aging.202958)
Supplement: Supplementary Table 1 [file aging-13-202958-s002.pdf]

SUPPLEMENTARY TABLE

Supplementary Table 1. Clinical pathological parameters of patients with OS in this study.

Characteristic	TARGET (with intact survival time)(n=85)	GSE21257 (n=53)
Age (years)		
<=18	66	35
>18	19	18
Survival status		
Alive	56	30
Dead	29	23
Gender		
Female	37	19
Male	48	34
Metastatic		
Metastatic	33	19
Non-metastatic	52	34
